# The State of the Art on Graphene-Based Sensors for Human Health Monitoring through Breath Biomarkers

**DOI:** 10.3390/s23229271

**Published:** 2023-11-19

**Authors:** Pedro Catalão Moura, Paulo António Ribeiro, Maria Raposo, Valentina Vassilenko

**Affiliations:** Laboratory for Instrumentation, Biomedical Engineering and Radiation Physics (LIBPhys-NOVA), Department of Physics, NOVA School of Science and Technology, NOVA University of Lisbon, Campus FCT-NOVA, 2829-516 Caparica, Portugal; pr.moura@campus.fct.unl.pt (P.C.M.); pfr@fct.unl.pt (P.A.R.); mfr@fct.unl.pt (M.R.)

**Keywords:** graphene, graphene-based sensors, biomarkers, exhaled air, lung cancer, gastric cancer, diabetes, sleep apnoea, chronic kidney diseases, asthma, chronic obstructive pulmonary disease

## Abstract

The field of organic-borne biomarkers has been gaining relevance due to its suitability for diagnosing pathologies and health conditions in a rapid, accurate, non-invasive, painless and low-cost way. Due to the lack of analytical techniques with features capable of analysing such a complex matrix as the human breath, the academic community has focused on developing electronic noses based on arrays of gas sensors. These sensors are assembled considering the excitability, sensitivity and sensing capacities of a specific nanocomposite, graphene. In this way, graphene-based sensors can be employed for a vast range of applications that vary from environmental to medical applications. This review work aims to gather the most relevant published papers under the scope of “Graphene sensors” and “Biomarkers” in order to assess the state of the art in the field of graphene sensors for the purposes of biomarker identification. During the bibliographic search, a total of six pathologies were identified as the focus of the work. They were lung cancer, gastric cancer, chronic kidney diseases, respiratory diseases that involve inflammatory processes of the airways, like asthma and chronic obstructive pulmonary disease, sleep apnoea and diabetes. The achieved results, current development of the sensing sensors, and main limitations or challenges of the field of graphene sensors are discussed throughout the paper, as well as the features of the experiments addressed.

## 1. Introduction

### 1.1. Biomarkers

The medical field is constantly seeking newer technologies and procedures that can enable successful, accurate and rapid diagnostics. The current diagnostic methodologies often rely on interventions that are invasive, painful, time-consuming and, at times, even unsafe to the patient, not to mention the commonly elevated costs and the lack of repeatability of the results [1,2]. To remedy these limitations, newer options have been studied regarding their suitability to diagnose pathologies and health conditions. Among those options, the detection, identification and quantification of biomarkers is gaining relevance [2,3].

A biomarker can be defined as any biological-borne molecule or organic feature, like the temperature, whose characteristics or detection can be indicative of the presence or development of a vast range of health conditions and pathologies [4]. In fact, the identification of biomarkers is often used to assess eventual health risks, to screen health state, to determine prognostics, to evaluate the response to treatments and even to evaluate the progress of a disease [5,6].

The most often studied biomarkers are compounds that are produced by the human organism as an outcome of the development or evolution of a specific pathology or health condition [7,8]. These molecules, once produced, interact with the biological tissues and are commonly released to the exterior of the organism through several body fluids. Breath, urine, faeces, perspiration, blood and even lacrimal fluid are examples of fluids that often carry molecules whose characteristics can make them act as biomarkers for diseases [9,10,11].

Among all the potential molecules studied as potential biomarkers, some of them can be linked to a vast range of conditions while others are studied regarding their exclusive relationship with a single disease. Acetone, for example, has been deeply addressed in the medical community since it can be used as a biomarker for at least 11 pathologies, namely sleep apnoea [12], malaria [13], lung cancer [14], gastric cancer [15], diabetes [16], cystic fibrosis [17], COVID-19 [18], colorectal cancer [19], chronic liver disease [20], chronic kidney disease (CKD) [21], and asthma [22]. Coincidently, the detection of isoprene in human emissions can equally be linked to 11 health conditions, which are chronic obstructive pulmonary disease (COPD) [23], chronic kidney disease [24], chronic liver disease [25], breast cancer [26], cystic fibrosis [27], COVID-19 [28], gastric cancer [29], diabetes [30], malaria [31], lung cancer [32], and sleep apnoea [12]. On the other hand, compounds like 2-acetylpyridine (chronic obstructive pulmonary disease [33]), ethanal (COVID-19 [28]), ethyl acrylate (cystic fibrosis [34]), 2-, 3- and 4-ethyltoluene (squamous cell cancer [35]), 5-ethyl-2-methylheptane (tuberculosis [36]), methyl butyrate (prostate cancer [37]) and many others are relatable with a single disease, as per the moment this paper was published.

Some of the mentioned biomarkers are well known and are even certified for medical use; nonetheless, many other organic-borne molecules still require deeper studies to fully assess their real dependence on a pathology or health condition and judge their suitability for diagnostic purposes. To do so, many scientific studies have been developed around several collection, separation and analysis techniques [38,39,40]. Among these, one can find works developed around ion mobility spectrometry [41], mass spectrometry [42], gas chromatography [43], and, as in for the scope of this paper, sensor array-based electronic noses built around several types of sensors [44], including graphene and graphene derivate-based sensors [45].

### 1.2. Graphene-Based Sensors

Graphene-based sensors have been largely explored regarding their vast range of applications, their adaptability to distinct scenarios and their proven results in the identification and quantification of specific molecules. Nonetheless, this exploration is only possible due to the characteristics of graphene whose excitability and sensitivity allow its application in the development of molecular electronic devices for sensing purposes.

Initially discovered by Geim et al. (2007) after peeling off the thin (atomic size) layers from pieces of graphite, graphene and graphene-derivative materials have played an important role in sensing-based scientific fields [46]. In fact, graphene is often described, at an atomic level, as the lightest, thinnest and strongest material, which presents hydrophobic behaviour, is aggregable in aqueous solutions, insoluble in organic solvents, and can be stable at temperatures as high as 200 °C [47]. Regarding its structure, graphene presents symmetric arrangements of bonds between carbon, which form a honeycomb pattern with a surface area of around 2600 m^2^/g [48].

Besides the mentioned features of graphene, it is worth saying that its major advantage is related to the fact that graphene characteristics can be easily altered if exposed to several scenarios. In fact, graphene can be chemically altered through π–π interactions or via electron transfer processes, for example, when exposed to scenarios rich in organic compounds whose functional groups are effortlessly attachable to graphene. In addition, graphene is an electrical conductor whose optical and electrical properties can be modified in several ways that include chemical, electrochemical and thermal options [47,49]. This reactivity and excitability of the graphene and graphene derivates materials make them ideal to be employed in several types of sensors, namely, optical fibre sensors, physical sensors, chemical sensors, electrochemical sensors, and wearable sensors [48,49].

**Figure 1:**
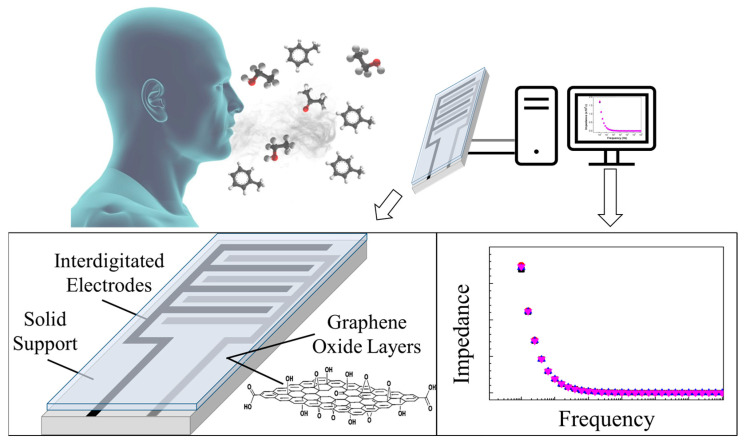
Schematic of a generic system based on graphene sensors for exhaled air analysis.

Evidently, each one of these types of sensors can be assembled through different techniques. One of the preparation methodologies of graphene-based sensors often used to analyse gaseous or volatile samples is the layer-by-layer technique. Here, alternated electrically charged thin films of polyelectrolytes are applied to a solid base containing interdigitated electrodes [50]. Examples of those polyelectrolytes are polyethyleneimine and, evidently, graphene oxide. The application consists of successive immersions of the base in the solutions. Once produced, the sensors can, then, be exposed to the volatile samples targeted during the experiment and, specifically, exhaled air samples. As addressed, there are several types of sensors and several ways of utilizing them. One of the options is reported by Moura et al. (2023) [50]. In their work, the authors registered the electrical response of the sensors through the impedance variance of the sensors when exposed to the target volatile organic compounds. Further details regarding the operating principle of the several types of graphene-based sensors can be found elsewhere [49]. Figure 1 schematizes a generic system based on graphene sensors to analyse exhaled breath through the impedance variation. Here, a graphene oxide-based sensor is exposed to the analytes emitted in breath and the impedance-frequency relationship is registered by a dedicated software. The graph illustrates the registered curves, in different colours, of two random compounds.

As addressed, each atom of graphene and graphene derivates is extremely sensitive and can easily be excited and react once exposed to a vast and varied range of scenarios that extend from environmental evaluation to health monitoring and assessment. These applications include examples as mixed as the study of industrially relevant alcohols [50], monitoring of volatile organic compounds emitted during extreme wildfires [51], identification of organic contaminants in aqueous matrices [52,53], detection of indoor air pollutants [54,55], and many other applications [56,57,58]. Among these topics, medical applications have been gaining relevance and importance in the field of graphene-based sensors, namely, the detection, identification and quantification of organic-borne biomarkers whose detection can represent an open window to the interior of the human organism and represent a valuable source of information on the health condition [45,59].

## 2. Graphene Sensors for Biomarker Detection

**Figure 2:**
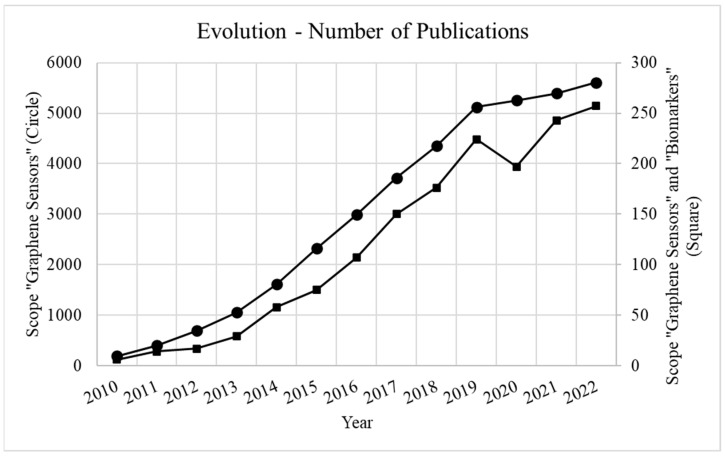
Number of scientific publications indexed in Web of Science for the past 10 years under the scope “Graphene Sensors” (circle markers) and the scope “Graphene Sensors and Biomarkers” (square markers).

Graphene-based sensors have gained relevance in the field of the detection, identification and quantification of compounds for medical purposes and, specifically, for diagnostics of pathologies via organic-borne biomarkers. In fact, the number of scientific publications on the topic has presents an evident positive rate of increase, as shown in Figure 2. Here, the number of publications indexed in one of the main scientific databases of publications, Web of Science, under the scope “Graphene Sensors” and the scope “Graphene Sensors and Biomarkers”, proves the increasing interest in this field and the growing dedication of the scientific community to the development and application of graphene-based sensors in the assessment of organic-borne biomarkers.

Having in mind all the facts and information previously addressed, one can deduce the pertinence of assessing the state of the art of the field. In this way, this review aims to share the current level of development in the field of graphene-based sensors for detecting, identifying, and quantifying volatile organic compounds present in the exhaled breath that, due to their organic origin, may act as biomarkers of specific pathologies and health conditions. To do so, the most relevant papers published under the scope mentioned above were considered and consulted for this review. The research was limited to the last 10 years of publication, i.e., 2012–2022. More than 3000 papers were initially gathered. Review papers were disregarded and the most relevant of the cohort were considered and reviewed in this article.

**Figure 3:**
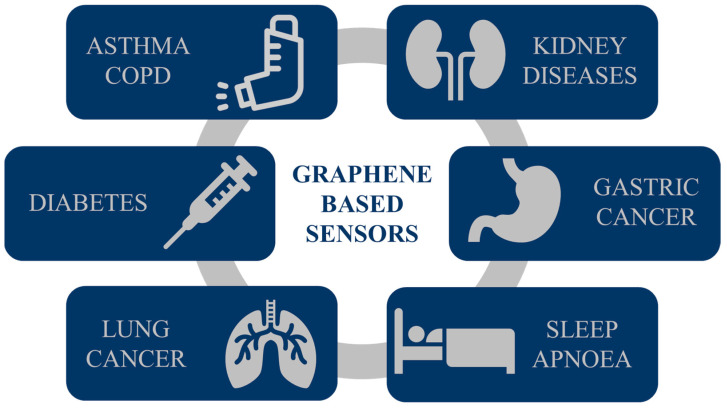
Six main pathologies are addressed under the scope of this review paper.

During the bibliographic search, one could realize that graphene-based sensors have been developed and employed to identify biomarkers in the breath of patients suffering from six specific pathologies. These six health conditions are represented in Figure 3. They are lung cancer, gastric cancer, chronic kidney diseases, respiratory diseases that involve inflammatory processes of the airways, like asthma and COPD), sleep apnoea and diabetes.

### 2.1. Asthma and COPD

Asthma, chronic obstructive pulmonary disease and other health conditions that involve inflammatory processes of the respiratory tract are among the most common and rather mortal pathologies worldwide. In fact, the World Health Organization (WHO) has reported a staggering number of 500,000 deaths directly caused by asthma, and 3 million deaths provoked by COPD worldwide every year [60,61]. These kinds of pathologies impose a very poor quality of life on the patients, whose state depends directly on the rapidness and effectiveness of the treatments [62,63]. In order to prescribe the proper treatment procedures, the physicians have to accurately diagnose the pathology in question; however, the current methodologies to do so are often time-consuming, expensive, invasive and very discomforting to the patients [64,65].

The detection, identification and quantification of volatile organic compounds in the breath of patients has gained relevance as a rapid, accurate, non-invasive, painless and low-cost procedure to identify inflammatory pathologies of the respiratory tract. Compounds such as acetone [22], decane [66], propanol [67], hexane [67], dodecane [66], ethylbenzene [68] and many others have been linked to the diagnosis of asthma when detected in exhaled air. A similar logic can be applied to the diagnostic of COPD via the identification of acetaldehyde [33], benzaldehyde [23], benzene [23], butanal [33], isoprene [23], isopropanol [33], limonene [69], nonanal [70], and many other analytes in breath.

Besides the potentialities of the biomarkers in breath, one of the current challenges to be surmounted is the lack of standardized procedures for the detection of those analytes. The field of graphene oxide-based sensors has positioned itself as one of the potential candidates to help overcome this issue. In fact, several works have been developed in the area [45,71].

Aiming to study one specific marker for inflammatory processes of the respiratory tract, nitrite, Gholizadeh et al. (2017) developed graphene oxide-based sensors that were later tested in two scenarios. Initially, the authors exposed the sensors to standard nitrile solutions prepared with different concentrations; then, the sensors were tested with samples of exhaled air condensate. The variation in the impedance of the sensors during the tests was assessed through impedance spectroscopy. In both scenarios, the authors were able to characterize the optimal operation conditions for the sensors, as well as successfully identify nitrite in both standard and exhaled air samples [72]. In a follow-up study, the authors were able to validate the results, showing the appropriateness of graphene sensors for medical applications and, in specific, for the identification of biomarkers for the diagnostic of inflammatory conditions of the respiratory tract [73].

The field of graphene oxide-based sensors has been making its contribution to the development of systems for the analysis of breath samples. The work of Kumar et al. (2020) is an example of that. The authors have developed an array of sensors based on graphene oxide composite to study three specific volatile compounds, ammonia, ethanol and the potential asthma biomarker, acetone. Standard samples of acetone were previously prepared with concentration levels of 1000 and 2000 ppm_v_. The developed sensors were capable of detecting, identifying and quantifying acetone successfully, proving their suitability for the eventual analysis of breath samples and asthma diagnosis [74].

Another well-known asthma biomarker is propanol [67]. This volatile organic compound has been studied through sensors of graphene oxide in several scientific studies. One of those works was developed by Samadi et al. (2021). The authors developed sensors capable of detecting propanol at room temperature by scattering thin layers of a specific nanocomposite, ZnO@SiO_2_/rGO. Then, the sensor was tested by being exposed to standard samples of propanol previously prepared with concentration levels ranging from 150 ppm_v_ to 450 ppm_v_. Propanol was successfully identified and quantified, proving the suitability of this kind of system for the assessment of biomarkers in breath [75].

Several works have reported a direct connection between the concentration levels of hexane in exhaled air, and the existence of inflammatory processes in the respiratory tract and, specifically, of asthma. Graphene-based sensors have been tested to assess their potential for hexane detection. Hussein et al. (2019) developed gas sensors by scattering thin layers of reduced graphene oxide (rGO) that were later used to detect three specific analytes, chloroform, ethanol and the potential asthma biomarker already mentioned, hexane. The tested volatile samples were previously prepared with known concentration levels of the target compounds. Hexane was successfully detected and quantified, proving that the system developed by the authors can eventually be tested and applied to real samples of exhaled air [76].

In order to detect the mentioned compounds in breath, some work has been performed regarding the graphene oxide sensing capacity. Murashima et al. (2016), for example, addressed the capacities of graphene-based sensors for the qualification and quantification of one of the aforementioned biomarkers, i.e., acetaldehyde. To do so, the authors developed the sensors by scattering thin films of graphene oxide that were later exposed to gaseous samples of acetaldehyde. The samples were prepared in 10 to 50% ratios between the target analyte and room air. All the samples were successfully detected, proving that the sensors developed by the authors can be utilized for clinical purposes [77].

As mentioned, another common biomarker of COPD is benzaldehyde. Coincidentally, Khan et al. (2015) studied this specific analyte with graphene oxide-based sensors. In fact, the authors developed an array of sensors that were later exposed to the standards of the target analytes. The authors were able to achieve sensitivity levels of ~1.2277 µAcm^−2^µM^−2^, proving the outstanding capacity of the developed gas sensors for the sensing of acetaldehyde [78].

Isoprene, a COPD biomarker often studied in breath, was successfully detected by an array of graphene oxide-based sensors developed by Chen et al. (2019). Authors were able to sense isoprene with limits of detection as low as 237 ppb_v_ in the volatile emissions of several types of food and, specifically, fruits. Besides that, considering the outstanding detection limits, their system proved their suitability and eventual applicability for exhaled air analysis and biomarkers identification [79].

A final example of a biomarker usually detected in breath and often linked to COPD is nonanal, as mentioned. With that in mind, Liu et al. (2019) developed an electronic nose based on chemoresistive sensors of graphene that was later used to analyse some specific analytes, including nonanal. The other studied volatile organic compounds were ethanol, 2-ethoxyethanol and ethylbenzene. The authors aim to develop more versatile and sensitive sensors; nonetheless, the achieved results leave no doubts regarding the pertinency of graphene-based sensors in the field of health monitoring and disease diagnosis [80].

Thousands of deaths occur every year directly due to respiratory pathologies and, specifically, due to asthma and chronic obstructive pulmonary disease. A considerable portion of these deaths could be avoided if a faster and more accurate diagnosis was possible. In this way, the scientific community has focused its efforts on developing electronic noses based on arrays of graphene sensors to detect organic-borne biomarkers that can act as biomarkers for the mentioned pathologies. As seen in the reviewed works, the community is working to develop more accurate, standardized systems to analyse human breath and the results already achieved are, undoubtedly, promising and worthy of attention.

Table 1 includes all the details on the procedures adopted in each one of the studies addressed in this section, including the target biomarkers and respective references on the Human Metabolome Database (HMDB), the considered populations, the developed sensors, and the respective bibliographic source.

### 2.2. Chronic Kidney Diseases

A fast and accurate diagnostic is especially mandatory in the case of chronic kidney diseases. This group of pathologies is responsible for leading thousands of people to hospitals every year due to direct complications of the pathology but also due to all the secondary consequences and comorbidities [81]. When identified in later stages of development CKD often results in mandatory haemodialysis treatment, acute renal failures and even life-threatening cardiovascular episodes [82,83].

Considering the aforementioned facts, it is crucial that newer, faster and more accurate procedures to diagnose CKD are developed. Electronic noses, whose working principle is based on the sensing capacities of graphene-based sensors, have been developed to tackle this challenge by qualifying and quantifying the analytes emitted in the breath of the patients that can act as biomarkers of CKD [84]. It is worth stating that several scientific studies have been published regarding this issue and whose focuses are given to known CKD biomarkers, namely, ammonia [85], acetone [86], ethanol [87], and isoprene [24], among others.

Aiming to assess the suitability of graphene-based sensors for the detection of volatile organic compounds often detected in breath, Lee et al. (2021) developed and validated reduced graphene oxide-based sensors and machine learning algorithms. The system was then tested with three specific volatile organic compounds, isopropanol, acetone and ammonia. As mentioned, ammonia is a common biomarker of chronic kidney diseases in the exhaled air so, with that in mind, authors exposed the sensors to several standard samples of the analyte, at specific concentration levels, and were able to identify it with accuracy levels above 90%. They were equally capable of differentiating health volunteers from chronic kidney disease patients, gathered in a synthetic cohort of volunteers, with accuracy levels above 20% [88].

Having the goal of detecting biomarkers of kidney pathologies in exhaled air, Majidi et al. (2021) developed and tested nitrogenated holey graphene sheet-based sensors. The target analytes of the authors were isoprene, pentanal, hexanal and heptanal, analytes commonly associated with kidney diseases. In this way, the authors studied the adsorption of these molecules on the surface of the developed sensors. They were able to successfully detect the analytes, proving the suitability of their system for further applications in the medical field and, in specific, for breath analysis [89].

Two specific volatile compounds commonly related to the diagnosis of chronic kidney diseases are acetone and ethanol. With that in mind, Tung et al. (2020) developed graphene-based chemoresistive sensors that were later used to detect specific volatile organic compounds, including acetone and ethanol. Other analytes equally relevant to the field of carcinogenic biomarkers were also tested; they are methanol, chloroform, acetonitrile and tetrahydrofuran. The developed array of sensors could detect the target compounds in limits of detection as low as 2.82 ppb_v_, outstanding results that undoubtedly show the suitability of graphene-based systems for lung cancer diagnosis via biomarkers in breath [90].

Acetone was also the target compound of the study developed by Choi et al. (2014). As stated by the authors, human exhaled air has tremendous advantages and a high potential to act as a valuable source of information about the organism and about specific pathologies and health conditions. In this way, and being aware of the relationship between the presence of acetone in breath and an eventual diagnosis of conditions like diabetes or gastric cancer, among others, the authors developed an array of sensors using WO_3_ hemitubes functionalized by graphene-based electronic sensitizers. To test the sensors’ performance, solutions of acetone were prepared with 1 ppm_v_ concentration. The sensors were then exposed to the solution and their response was assessed. A full detection of the target VOC was achieved with limits of detection as low as 100 ppb_v_ and with response times ranging between 11.5 and 13.5 s. The outstanding sensitivity and overall behaviour of the sensors leave no doubts about their suitability for real medical scenarios [91].

As mentioned above, CKD often result in mandatory haemodialysis treatments. Several procedures have been developed aiming to identify biomarkers in breath to assess the evolution and impact of the treatments in CKD patients [92,93].

Ammonia is a well-known volatile organic compound that has been studied regarding its direct connection to haemodialysis treatments. This compound occurs naturally in the human organism since it is produced by protein metabolism and is often excreted through urine. Nonetheless, it can traverse biological tissue and is often emitted through breath after being transported to the lungs via the circulatory system [94].

Aiming to identify ammonia in the exhaled air of patients undertaking haemodialysis treatment, Shahmoradi et al. (2021) fabricated graphene-based sensors, namely, sulfonate graphene-, graphene oxide- and reduced graphene oxide-based sensors. Then, the authors exposed the produced sensors to previously prepared gaseous samples of ammonia whose concentrations ranged from 0.5 ppb_v_ to 12 ppm_v_, similar concentration levels to the ones commonly found in the exhaled breath of haemodialysis patients. The system proved to be suitable for an accurate and sensitive detection of the target analyte in a non-invasive and painless way. The results prove the auspicious future of graphene-based sensors in the field of detection of biomarkers for the evaluation of the organism’s reaction to haemodialysis treatments and to diagnostic renal disease overall [95].

A final example of the work being developed in the field of graphene-based sensors for the detection, identification and quantification of biomarkers in breath is the study of Yabaş et al. (2023). To create the sensors, the authors synthesized 4-pyridynyl-oxadiazole tetrasubstituted zinc and cobalt phthalocyanine compounds that were, then, mixed with reduced graphene oxide to obtain hybrids, namely, rGO, rGO/ZnPc and rGO/CoPc. The performance of the array of sensors was tested against a total of five volatile compounds. Among these were ethanol and acetone, biomarkers for gastric cancer, and ammonia, a biomarker of haemodialysis treatment. Concentration levels ranging between 30 and 210 ppm_v_ were considered during the study, and response times of 190, 230 and 250 s were achieved, respectively, to rGO/CoPc, rGO/ZnPc and rGO-based sensors [96].

Chronic kidney diseases are serious conditions that affect the quality of life of the patients extremely, not only due to their own consequences but also to secondary comorbidities. All these consequences can be grandly diminished if the diseases are diagnosed in an initial stage of development. The detection of biomarkers in breath with graphene-based sensors has gained relevance in the past years as a rapid, accurate and non-invasive way of diagnosing CKD. The achieved results show that, once the procedures are standardized, all the target analytes are defined and the detection limits are improved, the field of graphene-based sensors and biomarkers will have a central role in modern medicine.

Table 2 includes all the details on the procedures adopted in each one of the studies addressed in this section, including the target biomarkers and respective references on HMDB, the considered populations, the developed sensors, and the respective bibliographic source.

### 2.3. Diabetes

Diabetes is among the most common pathologies worldwide, with notable incidence in developed countries. The WHO estimates that the number of patients suffering from diabetes will reach the humongous number of 350 million cases by the year 2030. Coincidentally, a considerable portion of the cases are diagnosed in a later stage of development, preventing a proper treatment and leading to relevant and, in some cases, life-threatening comorbidities [97,98].

The current procedures for monitoring blood glucose, besides being effective, are often invasive, complicated, and even expensive, so medical academia is constantly seeking new and non-invasive procedures that allow us to control diabetes accurately and rapidly [99]. In order to tackle this limitation, the scientific and medical communities have given full attention to the detection and identification of volatile organic compounds emitted in the breath that can act as biomarkers for the diagnosis of diabetes [100]. The main diabetes biomarkers are acetone [101], methanol [102], ethanol [102], isoprene [30], isopropanol [101] and others.

In order to help the medical field detect and identify the compounds emitted in breath, the scientific community investigating graphene-based sensors has placed their efforts in developing novel sensors, devices and even methodologies that allow a full characterization of the emitted analytes [103].

A direct example of the utility of graphene-based sensors can be found in the work of Kalidoss et al. (2019). In one of the first papers on the matter, authors developed gas sensors based on a ternary (graphene oxide, tin dioxide and titanium dioxide) nanocomposite for the detection of acetone in the breath of diabetic patients. Then, the authors exposed the developed sensors to several concentration levels of the analyte and fully characterized the behaviours of the sensors, namely, their response and recovery times, their ideal operating temperature, and even their sensitivity. The results achieved by the authors prove the promising future of this type of sensor [104].

During a second work, the authors developed and tested graphene-based chemoresistive sensors with the single purpose of detecting acetone in exhaled air samples. To do so, a prototypic device was developed around the array of sensors and used to analyse breath samples of 17 diabetic patients and 13 healthy volunteers. The authors claim that their system allows the differentiation among both groups of volunteers with an accuracy of over 60%, proving the suitability of graphene-base sensors for this type of application. Additionally, the authors intend to keep developing their prototype to the level of becoming a single lab-on-chip pocket device [105].

In a third work developed by the same research group, the authors used the data provided by the electronic nose developed around the array of graphene-based sensors, to develop an algorithm capable of increasing the already-good levels of accuracy of the sensors. During the study, exhaled air samples from a cohort of 60 volunteers (30 diabetic patients and 30 healthy subjects) were analysed with the device. The new approach to the data enabled the authors to increase the accuracy by up to 70% [106].

Acetone was also the biomarker of diabetes targeted by Thakur et al. (2022). An array of six sensors comprising hybridized graphene oxide field-effect transistors was developed by the authors specifically for the detection of acetone. To do so, the array was exposed to a dummy breath, i.e., a synthetically prepared breath whose purpose was to mimic the human exhaled air. The samples were prepared with synthetic air and specific portions of acetone whose concentration levels ranged between 400 ppb_v_ and 80 ppm_v_. Additionally, six other volatile compounds (formaldehyde, toluene, benzene, propanol, ethanol and methanol) were added to the mixture to act as interfering compounds during the analyses. Accuracy levels of 100% were achieved by the authors during the identification of acetone with the developed sensors [107].

In a direct application of graphene oxide nanosheets, Choi et al. (2014) focused their work on the sensing capacities of graphene oxide and developed gas sensors to detect the presence of acetone in the exhaled air of diabetic patients. Samples of acetone, previously prepared with concentration levels ranging between 1 and 5 ppm_v_, were used as target materials. The sensors were exposed to the samples and their behaviour was analysed. The authors state that the developed sensors enabled the detection of acetone with high levels of selectivity and limits of detection as low as 100 ppb_v_. This value proves the suitability of the described procedure to perform analyses of exhaled air, since the concentration of acetone in breath is often superior to 100 ppb_v_ [108].

In a very recent study, Sen et al. (2023) developed sensors based on reduced graphene oxide nanocomposites to detect acetone in the breath of a diabetic patient. The electrical response of the sensors exposed to the target analyte was studied through impedance spectroscopy. The capacity to detect concentration levels lower than 1 ppm_v_ and the excellent sensing response (response and recovery times of 10 and 30 s, respectively), prove the suitability of the assembled graphene-based sensors for the field of breath assessment [109].

Quantum resistive gas sensors to detect three specific analytes, acetone, methanol and ethanol, were prepared by Yempally et al. (2020) via the scattering of thin layers of graphene. To test the behaviour of the developed sensors when exposed to the target compounds, a mixture of the three compounds was previously prepared. The authors were capable of fully detecting and differentiating all the analytes with detection limits as low as 1 ppm_v_, exhibiting the potential of their system for real-scenario applications [110].

A final example of the suitability of graphene-based sensors for the detection of acetone in the breath of diabetic patients was developed by Rakkesh et al. (2022). The authors used a revolutionary microwave-assisted chemical reduction technique to extract layers of graphene from natural materials, specifically, coconut shells. Then, the synthesized nanostructure was evaluated regarding its capacity to sense acetone. Pure samples prepared with different concentration levels of the target compound were tested during the study for distinct conditions of analysis, namely, temperature and humidity conditions. The authors stated that the sensors enabled a successful detection of acetone and exhibited a response time/recovery time ratio of 1.11/41.25 s, proving the promising future of this field [111].

Interestingly, diabetes is among the health diseases most studied regarding the identification of biomarkers in exhaled air with graphene-based sensors. As for the other reviewed conditions, some challenges must be overcome, namely, the lack of standardized methodologies and the detection limit of the sensors that often fail to achieve concentration levels present in breath (low ppm_v_–ppb_v_). Once solved, this field will play a relevant role in the diagnosis and monitoring of diabetes in the near future.

Table 3 includes all the details on the procedures adopted in each one of the studies addressed in this section, including the target biomarkers and respective references on HMDB, the considered populations, the developed sensors, and the respective bibliographic source.

### 2.4. Gastric Cancer

The diagnostic of gastric cancer often involves a gastric endoscopy with a complementary biopsy and subsequent identification through histopathological analysis. As is known, this type of procedure is extremely invasive and leads the patient to scenarios of extreme discomfort. Additionally, the results often require some time to be available causing a delay in the treatment procedures. In this way, the development of non-invasive, rapid and accurate techniques for gastric cancer diagnosis is required to allow proper treatment of the disease as rapidly as possible [112,113].

Aiming to accelerate the diagnosis of the pathology, the identification of gastric cancer biomarkers in breath has gained pertinency [114]. One can find several works whose scopes were dedicated to the identification of biomarkers; they are acetic acid [115], acetone [116], 2-butanone [117], isoprene [115], propanal [115], phenyl acetate [116], furfural [118], toluene [119], and many others.

To overcome the challenges of detection and analysis of volatile organic compounds, the field of electronic noses based on graphene oxide sensors has played an important role. A considerable number of works have been published regarding the capacity of graphene for the sensing of breath biomarkers [120].

A total of 14 volatile organic compounds present in exhaled air samples were studied by Chen et al. (2016), during their search for gastric cancer biomarkers. To do so, the authors developed gas sensors based on the scattering of graphene oxide thin films that were later exposed to a cohort of 200 simulated breath samples of healthy volunteers (56 subjects), early-stage gastric cancer (55 subjects) and advanced-stage gastric cancer (89 subjects). The authors were able to successfully differentiate the samples with levels of sensitivity above 80%. As mentioned, a total of 14 biomarkers were studied during the study, namely, 2-methylhexane, 2-methylpentane, 3-methylpentane, dodecane, tetradecane, menthol, phenyl acetate, hexanol, pivalic acid, 3-methylhexane, 2,3-dimethylpentane, hexane, isoprene and acetone [116].

Acetic acid was one of the analytes targeted by Moura et al. (2023) in their recent study on volatile compounds. The authors developed graphene oxide sensors based on thin films scattering for the purpose of sensing four specific analytes, methanol, isopropanol, ethanol and acetic acid. Then, the authors exposed the sensors to samples of acetic acid ranging from 24 to 120 ppm_v_ and successfully detected and quantified all the samples. The entire procedure and, specifically, the resolution of 0.04 ppm_v_ achieved by the sensors prove their eventual applicability to analyse exhaled air samples [50].

Another example of the suitability of graphene oxide-based sensors for breath assessment is the work of Jia et al. (2022). Aiming to identify and quantify acetone in breath, the authors synthesized Fe_3_O_4_/rGO composites through a simple hydrothermal reaction that was later used to develop the sensors. Then, the array was exposed to samples of acetone previously prepared at several concentration levels (maximum of 800 ppm_v_). The main goal of the authors was to identify acetone as a diabetes biomarker in breath, and that was completely achieved; nonetheless, considering that acetone can equally act as gastric cancer biomarkers, the results are auspicious and also promising to this field of research [121].

As mentioned, acetone, isoprene and toluene are well-studied analytes often detected in breath, whose levels of concentration can be indicative of carcinogenic pathologies and, specifically, gastric cancer. Being aware of this fact, Tombel et al. (2021) decided to assess the suitability of graphene-based sensors to study the mentioned analytes. In this way, authors integrated reduced graphene oxide on Ti/Pt interdigitated electrode deposited on a SiO_2_/Si substrate that was later exposed to acetone, isoprene and toluene. The standard solutions used as samples were previously prepared with concentration levels in the ppm_v_ range (up to 6 ppm_v_). The sensors showed outstanding capacities for detecting all the compounds; in fact, the identification of acetone, toluene and isoprene was achieved with R-squared values of 0.9312, 0.0093 and 0.9388 [119].

The diagnostic of gastric cancer, as mentioned in the initial portion of this chapter, often requires invasive and painful procedures. The field of graphene-based sensors has made a contribution to overcoming these issues. The detection of biomarkers in the breath of gastric cancer patients, in this way, has grown as a potential solution. Once overcome limitations like the lack of standardized procedures, the lack of portability of the developed systems and the full identification of the target analytes, graphene-based sensors can be, without doubt, a useful tool for the medicine of the future.

Table 4 includes all the details on the procedures adopted in each one of the studies addressed in this section, including the target biomarkers and respective references on HMDB, the considered populations, the developed sensors, and the respective bibliographic source.

### 2.5. Lung Cancer

Due to its direct connection to the respiratory system, lung carcinoma has been deeply studied regarding the possibility of a faster, more accurate, non-invasive and painless diagnostic that would allow a proper and effective treatment in expedited time. This is, in fact, one of the main necessities of current medicine, accurately and rapidly diagnosing lung cancer [122,123]. The high levels of incidence allied to the mortality of the pathology make it one of the most concerning conditions worldwide. In fact, every year, more than 2 million new cases are diagnosed on the planet [124].

Several approaches, procedures and methodologies have been used to detect lung cancer. The detection of biomarkers in the exhaled air is one of them [125,126]. One can list several analytes often linked to pulmonary carcinogenic conditions; they are heptane [127], hexanal [128], pentane [32], 2-butanone [129], furan [130], decane [14], acetone [32], isoprene [131], ethanol [132], or even formaldehyde [133], among many others. To supplant the issues of analysing and identifying the volatile analytes emitted in breath, the field of graphene oxide-based sensors has given its contribution by developing innovative electronic noses based on the sensing capacities of graphene [134]. In fact, the literature provides some examples of that contribution.

One of the main examples of the utilization of systems based on graphene-oxide sensors for the analysis of breath in the search for lung cancer biomarkers is the work of Emam et al. (2020). A total of nine volatile compounds (butyraldehyde, tetrahydrofuran, acetonitrile, heptane, hexanal, benzene, 2-butanone and furan) were analysed with electrochemical gas sensors developed via the deposition of thin layers of graphene and Prussian blue on a chromium-modified silicon substrate. To do so, the authors developed an entire system around the array of sensors that registered the resistance variation on the sensors once exposed to the target analytes. These variations were, then, saved for consultation in a mobile application specifically developed for this project. The tests were performed using pure standards of the nine biomarkers, a procedure that allowed the authors to certify the correct operability and suitability of their system for clinical purposes [135].

Graphene-based sensors were also the basis of the analytical technique employed by Shanmugasundaram et al. (2022) to study two specific compounds, decane and heptane. These compounds are well-known biomarkers often detected in the breath of lung cancer patients [6]. To detect them, the authors developed a methodology based on SnO_2_ nanospheres and a reduced-graphene-oxide-incorporated SnO_2_ nanocomposite. Then, the sensors were exposed to standard samples of the target gases, allowing the authors to fully assess the behaviour of the developed system during the exposure. The sensors proved to be capable of detecting concentration levels as low as 1 ppm_v_ and the authors intend to test them in real samples of breath [136]. Coincidently, decane was also studied by Zhang et al. (2016). To do so, the authors developed surface acoustic wave gas sensors coated with thin films of graphene oxide and exposed them to standard samples of the target analyte. The authors were able to detect this well-known biomarker with sensitivity levels as low as 0.2 ppm_v_, proving the suitability of the technique for breath analyses [137].

An electronic nose was developed by Chen et al. (2020) to analyse breath samples and detect potential biomarkers for lung cancer in human exhaled air. The electronic nose consisted of an array of chemoresistive graphene oxide-based sensors whose resistance variation was assessed when exposed to real samples of breath. A cohort of 108 subjects was considered for the study and, with the developed E-nose the authors could differentiate both groups with sensitivity and specificity levels of 95.8% and 96.0%, respectively. Acetone, isoprene and ammonia were the analytes addressed as potential lung cancer biomarkers during the study [138].

Acetone and ethanol were equally addressed as potential biomarkers for lung cancer diagnosis through breath analysis by Sánchez-Vicente et al. (2020). Here, the authors used graphene-doped tin oxide nanofibres and nanoribbons as gas sensors to analyse previously developed synthetic samples of breath. The synthetic samples were prepared with known concentrations of acetone and ethanol, ranging between 0.5 and 4 ppm_v_, to simulate both healthy individuals and pathological volunteers. The developed system could successfully differentiate both groups with outstanding capacity, proving that the authors can now test it using real samples of exhaled air [139].

A final biomarker often linked to the diagnosis of lung cancer is formaldehyde, as mentioned. In order to assess its real suitability for helping to diagnose lung cancer in a rapid, painless, noninvasive and accurate way, Shanmugasundaram et al. (2022) developed an electronic nose whose operating principle was based on reduced graphene oxide superstructures. The authors simulated exhaled air samples of healthy people and lung cancer patients by preparing solutions of formaldehyde with specific concentration levels, namely, 49 ppb_v_ to healthy samples and 83 ppb_v_ to pathological samples. Once exposed to the cohort, the developed sensors were capable of fully differentiating between both groups with pinpoint accuracy. This outstanding evidence proves the suitability of graphene oxide-based systems to diagnose lung cancer through biomarkers in breath [140].

As addressed, several works have dedicated their scope to the development of graphene-based sensors for the detection of biomarkers in the breath of lung cancer patients. Nonetheless, several challenges have to be overcome, namely, the improvement in the detection systems, the standardization of the collection and analysis procedures, and the improvement in the detection limits and sensitivity of the sensors. All in all, the results already achieved prove that the future of lung cancer assessment through graphene sensors is auspicious and should not be disregarded.

Table 5 includes all the details on the procedures adopted in each one of the studies addressed in this section, including the target biomarkers and respective references on HMDB, the considered populations, the developed sensors, and the respective bibliographic source.

### 2.6. Sleep Apnoea

It is estimated that a staggering number of 936 million adults suffer from sleep apnoea worldwide [141]. This pathology is characterized by profound alterations in the breathing rhythm, i.e., variations between breathing and non-breathing periods during sleep due to the collapse of the airways. The patients are, then, exposed to a vast range of commodities and consequences from this successive oxygenation interruption [142,143].

As with other respiratory pathological conditions, sleep apnoea has been studied regarding eventual biomarkers emitted in the breath that can lead to a rapid, accurate and non-invasive diagnosis. The main analytes often linked to sleep apnoea are acetone [12], decanal [12], heptane [12], hexane [12], nonane [12], octane [12], β-pinene [144], toluene [12], and p-xylene [12], among some others. Due to the current challenges in the detection of these analytes in samples of breath, several procedures have been developed around an array of sensors based on the sensing capacities of a specific nanocomposite, graphene [145].

As mentioned above, toluene is one of the biomarkers often found in the breath of sleep apnoea patients. Coincidently, toluene was one of the volatile organic compounds studied by Casanova-Chafer et al. (2021). To do so, the authors based their work on the sensing capacities of nanohybrid composites comprising graphene loaded with perovskite nanocrystals. Then, the array of sensors was exposed to the standard solutions of toluene prepared in concentration levels of 2, 4, 6 and 8 ppm_v_. The behaviour of the sensors was assessed for all four situations, and they left no doubts about their suitability for the detection of gaseous samples of toluene and, consequently, their applicability for exhaled-air study [146].

Once again, acetone has shown itself as a common compound commonly present in the exhaled air and often linked to health conditions. Sleep apnoea is no exception cit. With that in mind, Sen et al. (2021) developed an array of sensors based on ZnO-SnO_2_ nanocomposites decorated with reduced graphene oxide. Then, the authors tested the sensors regarding their sensing capacities by exposing them to solutions of acetone prepared at concentration levels ranging between 1 and 10 ppm_v_. The outstanding accuracy of 91% and detection limits of 0.675 ppm_v_ prove not only the perfect capacity for volatile acetone detection but also the suitability for the identification of biomarkers in breath [147].

Aiming to develop a wearable sensor for breath monitoring, Caccami et al. (2017) turned to the sensing capacities of graphene and developed graphene oxide-based sensors capable of adhering to the skin and continuously monitoring the exhaled air of the volunteer. The authors focused their work not on the variation of the sensors’ resistance when exposed to specific analytes, but on the respiratory (inspiration/expiration) cycles to assess conditions like apnoea and tachypnoea [148]. In a follow-up study, Caccami et al. (2018) were able to test their graphene-based sensors in 10 volunteers who were asked to reproduce a predefined set of normal and abnormal respiratory rhythms. Again, authors were able to prove the suitability of sensors based on graphene to fully characterize the exhaled breath and, consequently, assess diseases and health conditions like apnoea [149].

Isopropanol, also known as isopropyl alcohol, is another biomarker of sleep apnoea whose detection has been studied with graphene-based sensors. An example of that is the work of Ray et al. (2022). Aware that the breathomics is the future of non-invasive, painless, accurate and rapid medicine, the authors developed graphene-based sensors that were then used to analyse real breath samples. The breath samples were collected and spiked with concentrations of 0.5 ppm_v_ of well-known volatile organic compounds: acetone, acetaldehyde, butanediol, cyclohexanone, decane, ethanol, methanol, octane, styrene, propyl benzene and, as previously addressed, the sleep apnoea biomarker, isopropanol. Outstanding levels of accuracy (ranging between 92.8 and 96%). Considering the achieved results, there is no doubt about the total suitability of graphene-based sensors for the identification of biomarkers in breath [150].

Without question, the most common pathology in the world from six reviewed in this paper, sleep apnoea is responsible for the lack of life quality of almost 1 billion people. Aiming to improve the life quality of all these people, academia has focused on developing newer electronic noses based on arrays of graphene sensors, which allow the diagnosis and monitoring of sleep apnoea in an accurate, rapid, non-invasive, painless and low-cost way. As seen throughout the chapter, these systems still present some challenges to overcome, namely, the lack of standards for the collection and analysis of breath samples, the lack of detection limit capable of equalizing the concentration levels often found in exhaled air, and evidently, the certification of all these technologies for real medical applications. Nonetheless, the future looks promising, and modern medicine will rely, once again, on the achievements of academia.

Table 6 includes all the details on the procedures adopted in each one of the studies addressed in this section, including the target biomarkers and respective references on HMDB, the considered populations, the developed sensors, and the respective bibliographic source.

## 3. Conclusions

Medicine of the future will deeply rely on modern methods of diagnosis that allow the identification of pathologies and health conditions in faster, more accurate, painless and non-invasive ways, to the detriment of the current procedures that often involve risks, pain, long periods of waiting and failure to offer repeatable results. The detection, identification and quantification of biomarkers emitted in the exhaled air of patients suffering from the target pathologies is a field of growing interest and one whose past results prove its pertinency.

In order to fully use the potentialities of breath biomarkers, accurate, standardized and sensitive detection systems are required. Nonetheless, not all the analytical techniques and electronic noses available have the necessary features to analyse such a complex matrix as it is the human breath. With this in mind, the scientific community has devoted considerable efforts to developing new systems based on the sensing capacities of a specific nanocomposite, graphene, for the qualification and quantification of organic-borne biomarkers.

The present review aimed to gather the most relevant published papers under the scopes “Graphene-based sensors” and “Biomarkers”, in order to assess the state of the art of the field, identify the developments achieved during the past years, and spot the main pathologies under exploration regarding the suitability of graphene sensors to detect biomarkers. A total of 3000 publications were initially considered and once reduced to the most relevant ones, one could identify a total of six main areas of work. They are lung cancer, gastric cancer, diabetes, sleep apnoea, chronic kidney diseases and respiratory conditions like asthma and chronic obstructive pulmonary disease.

Considering all the reviewed works, one can state that impressive results have been achieved in the field of graphene sensors for breath analysis. A considerably elevated number of systems have been successfully developed and tested both in laboratory scenarios and with real samples of exhaled air. Some of the sensors accurately detected the target analytes with detection limits comparable to the concentration levels often found in breath (low ppm_v_–ppb_v_). Some could provide repeatable results from the data produced during the measurements. All these facts prove the auspicious and promising future of the field.

Besides the advantages and positive results, some issues must be overcome in order to reinforce the relevancy of this field. The lack of standardized procedures and, consequently, the different results achieved by independent groups is one of those limitations. This deficiency in the standardization of the methodologies and systems employed for the fabrication of sensors and consequent detection of biomarkers leads to the impossibility of comparing and combining the data reported. Then, the complexity of the hardware utilized in the production of the systems is equally challenging since it often prevents on-site clinical studies and large medical trials. The reduced number of trials and, specifically, experiments with graphene-based sensors and real humans are another issue that must be assessed and overcome.

Still under the topic of limitations, this review paper proves that only some of the current systems exhibit detection limits that achieve the desired levels. Compounds in breath are often present in breath at low ppm_v_ and even ppb_v_ levels, and it is evident that not all the systems could reach such levels in order to be tested against biological samples in real scenarios.

Finally, one can conclude that another major challenge of this field is directly related to the biomarkers themselves. As noticeable throughout the review, the analytes are often linked to more than one pathology. An evident example of that is acetone, which has been studied regarding its suitability to act as a biomarker of all the eight pathologies here addressed, namely, asthma and COPD, CKD, diabetes, gastric and lung cancer, and sleep apnoea. This lack of specificity of the detected biomarkers prevents their contemporary utilization for medical purposes since further studies are required to fully assess how their presence and concentration levels can be related to a specific pathology.

One can foresee that when all these issues are fixed, the graphene-based sensors and the detection of organic-borne biomarkers will, undoubtedly, play a relevant role in the medicine of the future and will help improve medical care worldwide.

## Figures and Tables

**Table 1:** Details on the procedures adopted in each one of the studies addressed in the section “Asthma and COPD”, including the target biomarkers, the considered population, the developed sensors and the respective bibliographic source.

Target Biomarker		Population		Sensors				References
Name	HMDB	Target	Notes	Sensitivity	Detection Limits	Response/Recovery Time	Accuracy	
Nitrite	HMDB0002786	Human subjects	Exhaled air condensate	–	–	–	100%	[72]
Nitrite	HMDB0002786	Human subjects	Exhaled air condensate	–	–	–	100%	[73]
Acetone	HMDB0001659	Standard solutions	Two known concentrations	–	1000–2000 ppm_v_	100/–s	–	[74]
Propanol	HMDB0000820	Standard solutions	Known concentrations (150–450 ppm_v_)	300 ppm_v_	4 ppm_v_	156.85	95%	[75]
Hexane	HMDB0029600	Standard solutions	Solutions of three compounds	–	–	–	–	[76]
Acetaldehyde	HMDB0000990	Standard solutions	Gaseous samples	1.012–1.043	–	30–70/45–85 s	–	[77]
Benzaldehyde	HMDB0006115	Standard solutions	Gaseous samples	1.2277	0.03 nM	10/–s	–	[78]
Isoprene	HMDB0253673	Standard solutions	Gaseous samples (5–160 ppm_v_)	–	237 ppb_v_	–	–	[79]
Nonanal	HMDB0059835	Standard solutions	Binary mixture	–	25 ppm_v_	61–200/97–416 s	–	[80]

**Table 2:** Details on the procedures adopted in each one of the studies addressed in the section “Chronic Kidney Diseases”, including the target biomarkers, the considered population, the developed sensors and the respective bibliographic source.

Target Biomarker		Population		Sensors				References
Name	HMDB	Target	Notes	Sensitivity	Detection Limits	Response/Recovery Time	Accuracy	
Ammonia	HMDB0000051	Standard solutions + Synthetic Breath	24 Levels of concentration	–	–	–/–	91.7%	[88]
Isoprene	HMDB0253673	Standard solutions	Known concentrations	–	–	–/–	–	[89]
Pentanal	HMDB0031206							
Hexanal	HMDB0005994							
Heptanal	HMDB0031475							
Acetone	HMDB0001659	Standard solutions	Gaseous samples	–	2.82 ppb_v_	–/–	–	[90]
Ethanol	HMDB0000108							
Acetone	HMDB0001659	Standard solutions	Solutions of 1 ppm_v_	1.7	100 ppb_v_	11.5–13.5/–s	–	[91]
Ammonia	HMDB0000051	Standard solutions	Known concentrations	–	0.2 ppb_v_–12 ppm_v_	48/234 s	–	[95]
Acetone	HMDB0001659	Standard solutions	Known concentrations (30–210 ppm_v_)	–	82 ppb_v_	190–250/–s	–	[96]
Ethanol	HMDB0000108							
Ammonia	HMDB0000051							

**Table 3:** Details on the procedures adopted in each one of the studies addressed in the section “Diabetes”, including the target biomarkers, the considered population, the developed sensors and the respective bibliographic source.

Target Biomarker		Population		Sensors				References
Name	HMDB	Target	Notes	Sensitivity	Detection Limits	Response/Recovery Time	Accuracy	
Acetone	HMDB0001659	Standard solutions	–	6.28	0.25–30 ppm_v_	–/–	–	[104]
Acetone	HMDB0001659	Human subjects	30 Volunteers: 17 diabetic patients and 13 healthy individuals	5.66	–	10/12 s	60%	[105]
Acetone	HMDB0001659	Human subjects	60 Volunteers: 30 diabetic patients and 30 healthy individuals	–	0–3 ppm_v_	–/–	70%	[106]
Acetone	HMDB0001659	Synthetic breath	Four distinct solutions	0.5–3.5	400 ppb_v_–80 ppm_v_	–/–	100%	[107]
Acetone	HMDB0001659	Standard solutions	–	10.0	>100 ppb_v_	–/–	–	[108]
Acetone	HMDB0001659	Human subjects	–	7.8	<1 ppm_v_	10/30 s	–	[109]
Acetone	HMDB0001659	Standard solutions	Mixture of known concentrations	–	1–100 ppm_v_	–/–	–	[110]
Methanol	HMDB0001875							
Ethanol	HMDB0000108							
Acetone	HMDB0001659	Standard solutions	–	–	1–5 ppm_v_	1.11/41.25 s	–	[111]

**Table 4:** Details on the procedures adopted in each one of the studies addressed in the section “Gastric Cancer”, including the target biomarkers, the considered population, the developed sensors and the respective bibliographic source.

Target Biomarker		Population		Sensors				References
Name	HMDB	Target	Notes	Sensitivity	Detection Limits	Response/Recovery Time	Accuracy	
Acetic acid	HMDB0000042	Standard solutions	Three known concentrations	30.3	0.04 ppm_v_	–	–	[50]
Acetone	HMDB0001659	Standard solutions	Concentrations up to 6 ppm_v_	–	1 ppm_v_	15/75	–	[119]
Isoprene	HMDB0253673							
Toluene	HMDB0034168							
2-Methylhexane	HMDB0245230	Synthetic breath	Mixture of 14 compounds at known concentrations	83%	–	–/–	92%	[116]
2-Methylpentane	HMDB0061884							
3-Methylpentane	HMDB0061885							
Dodecane	HMDB0031444							
Tetradecane	HMDB0059907							
Menthol	HMDB0003352							
Phenyl acetate	HMDB0040733							
Hexanol	HMDB0012971							
Pivalic acid	HMDB0041992							
3-Methylhexane	HMDB0245932							
2,3-Dimethylpentane	HMDB0245455							
Hexane	HMDB0029600							
Isoprene	HMDB0253673							
Acetone	HMDB0001659							
Acetone	HMDB0001659	Standard solutions	Concentrations up to 800 ppm_v_	–	35 ppm_v_	–	–	[121]

**Table 5:** Details on the procedures adopted in each one of the studies addressed in the section “Lung Cancer”, including the target biomarkers, the considered population, the developed sensors and the respective bibliographic source.

Target Biomarker		Population		Sensors				References
Name	HMDB	Target	Notes	Sensitivity	Detection Limits	Response/Recovery Time	Accuracy	
Butyraldehyde	HMDB0003543	Standard solutions	Known concentrations	–	1–20 ppb_v_	–/–	–	[135]
Tetrahydrofuran	HMDB0303508							
Acetonitrile	HMDB0061869							
Heptane	HMDB0031447							
Hexanal	HMDB0005994							
Benzene	HMDB0001505							
Pentane	HMDB0029603							
2-Butanone	HMDB0000474							
Furan	HMDB0013785							
Decane	HMDB0031450	Standard solutions	Known concentrations	–	1 ppm_v_	15/90	–	[136]
Heptane	HMDB0031447					19/48		
Decane	HMDB0031450	Standard solutions	Known concentrations	–	0.2 ppm_v_	28/37 s	–	[137]
Acetone	HMDB0001659	Human subjects	108 Volunteers: 48 healthy individuals and 60 lung cancer patients	–	0.05–10 ppm_v_	60/180 s	96%	[138]
Isoprene	HMDB0253673							
Ammonia	HMDB0000051							
Ethanol		Synthetic breath	Known concentrations	–	0.5 ppm_v_	50/60 s	–	[139]
Acetone	HMDB0001659							
Formaldehyde	HMDB0001426	Synthetic breath	Lung cancer samples with 83 ppb_v_ and healthy samples with 49 ppb_v_	100 ppb_v_	10 ppb_v_	–	–	[140]

**Table 6:** Details on the procedures adopted in each one of the studies addressed in the section “Sleep Apnoea”, including the target biomarkers, the considered population, the developed sensors and the respective bibliographic source.

Target Biomarker		Population		Sensors				References
Name	HMDB	Target	Notes	Sensitivity	Detection Limits	Response/Recovery Time	Accuracy	
Toluene	HMDB0034168	Standard solutions	Concentrations of 2, 4, 6 and 8 ppm_v_	0.5	–	–/–	–	[146]
Acetone	HMDB0001659	Standard solutions	Concentrations of 1–10 ppm_v_	–	0.675 ppm_v_	10/100	91%	[147]
Isopropanol	HMDB0000863	Human subjects	Breath spiked with 0.5 ppm_v_ of several compounds	–	0.5 ppm_v_	-	92.8–96%	[150]

## Data Availability

Not applicable.
